# Functional analysis of the translation factor aIF2/5B in the thermophilic archaeon *Sulfolobus solfataricus*

**DOI:** 10.1111/j.1365-2958.2007.05820.x

**Published:** 2007-08-01

**Authors:** Enzo Maone, Michele Di Stefano, Alessandra Berardi, Dario Benelli, Stefano Marzi, Anna La Teana, Paola Londei

**Affiliations:** 1 Dpt. of Biotecnologie Cellulari ed Ematologia, Università di Roma Sapienza Viale Regina Elena 324 Roma, Italy.; 2 Istituto di Biochimica, Università Politecnica delle Marche Via Ranieri Ancona, Italy.; 3 DIBIFIM, Università di Bari, Piazzale Giulio Cesare Bari, Italy.; 4 Institut for de Biologie Moleculaire et Cellulare CNRS 67084 Strasbourg Cedex, France.

## Abstract

The protein IF2/eIF5B is one of the few translation initiation factors shared by all three primary domains of life (bacteria, archaea, eukarya). Despite its phylogenetic conservation, the factor is known to present marked functional divergences in the bacteria and the eukarya. In this work, the function in translation of the archaeal homologue (aIF2/5B) has been analysed in detail for the first time using a variety of *in vitro* assays. The results revealed that the protein is a ribosome-dependent GTPase which strongly stimulates the binding of initiator tRNA to the ribosomes even in the absence of other factors. In agreement with this finding, aIF2/5B enhances the translation of both leadered and leaderless mRNAs when expressed in a cell-free protein-synthesizing system. Moreover, the degree of functional conservation of the IF2-like factors in the archaeal and bacterial lineages was investigated by analysing the behaviour of ‘chimeric’ proteins produced by swapping domains between the *Sulfolobus solfataricus* aIF2/5B factor and the IF2 protein of the thermophilic bacterium *Bacillus stearothermophilus*. Beside evidencing similarities and differences between the archaeal and bacterial factors, these experiments have provided insight into the common role played by the IF2/5B proteins in all extant cells.

## Introduction

Initiation is the rate-limiting step of protein synthesis, during which the ribosome, aided by several proteins called initiation factors, lands on the initiation codon of a mRNA and establishes the correct reading frame for decoding. This phase of translation presents relevant differences in the primary domains of cell descent – the bacteria, the archaea and the eukaryotes – as regards both the mechanism and the components involved. Bacterial ribosomes can recognize directly the translation initiation region (TIR) by means of the Shine–Dalgarno (SD)–anti-SD interaction; just three initiation factors participate in the process (Gualerzi and Pon, 1990). In the eukaryotes, mRNA/ribosome interaction and the selection of the correct initiation codon are believed to take place by a ‘scanning’ mechanism, whereby the 40S subunit binds at, or near, the capped 5′ end of the mRNA and moves in a 3′ direction until the initiator AUG – usually the first available one – is found (Kozak, 1989). This process requires the assistance of over a dozen protein factors, several of which are involved in cap recognition and in ATP-driven mRNA unwinding while others interact with the ribosome and/or the mRNA helping the correct selection of the initiation codon, the joining of the ribosomal subunits and the formation of the first peptide bond (Merrick, 1992). Recent studies on initiation of protein synthesis in the third kingdom of life, the archaea, have revealed ‘hybrid’ features somewhat intermediate between bacterial and eukaryal ones. Like bacteria, the archaea have polycistronic mRNAs often endowed with SD motifs for ribosome recruitment to the translation initiation sites (Condo *et al*., 1999; Sartorius-Neef and Pfeifer, 2004). However, the set of putative archaeal translation initiation factors is more extended than the bacterial one, as it includes at least six proteins whose primary sequences have the greatest homology with eukaryal initiation factors. Among these there are homologues of the three subunits of eIF2, the factor that interacts with met-tRNAi, and homologues of eIF1, eIF6, eIF5A, eIF1A and eIF5B. The latter three proteins are actually universal translation factors, as homologues thereof are also encountered in bacteria, respectively, called EF-P, IF1 and IF2 (Kyrpides and Woese, 1998).

The universal factor IF2/eIF5B is a very interesting protein that has received of late a great deal of attention. In spite of its universal conservation, the factor presents marked functional divergences in the primary domains. Bacterial IF2 is the central player in translation initiation: it interacts with GTP and fMet-tRNAi, adapting the latter into the ribosomal P site. However, in the eukarya the Met-tRNAi binding factor is the trimeric protein eIF2, none of whose subunits is related to bacterial IF2. The eukaryal IF2 homologue eIF5B has been implicated in promoting subunit joining in a late initiation step (Pestova *et al*., 2000; Shin *et al*., 2002). In archaea the Met-tRNAi-binding protein is the eIF2 homologue a/eIF2 (Yatime *et al*., 2004; Pedulla *et al*., 2005), while the function of the eIF5B homologue (henceforth termed aIF2/5B) is as yet unclear. The finding that aIF2/5B from *Methanocaldococcus jannaschii* can partially replace the loss of eIF5B *in vivo* (Lee *et al*., 1999) points to an at least partial overlapping of eIF5B and aIF2/5B functions. However, the role in protein synthesis of the archaeal factor remains to be analysed experimentally, also in view of answering several important questions about the evolution of translation. Given the universal conservation of the IF2-like factors, which was their (presumably essential) function in ancestral translation initiation? Why, and when, the tRNAi binding factor diverged in the primary domains?

In the present work we have performed a detailed analysis of the function of aIF2/5B in the thermophilic archaeon *Sulfolobus solfataricus*. The results revealed that the protein is a ribosome-dependent GTPase which strongly stimulates the binding of Met-tRNAi to the ribosomes even in the absence of other factors. In agreement with this finding, aIF2/5B enhances the translation of both leadered and leaderless mRNAs when expressed in a cell-free protein-synthesizing system. Moreover, we investigated the degree of functional conservation of the IF2-like factors in the archaeal and bacterial lineages by analysing the behaviour of ‘chimeric’ proteins produced by swapping domains between the *S. solfataricus* aIF2/5B factor and the IF2 protein of the thermophilic bacterium *Bacillus stearothermophilus*.

## Results

### Cloning of the *S. solfataricus* IF2/5B gene

The *S. solfataricus* IF2/5B gene was amplified by PCR and inserted into an expression vector (pRSETB) that added a tag of six histidines to the N-terminus of the protein and whose transcription was directed by the promoter of phage T7. The correctness of the insertion and of the nucleotide sequence of the PCR fragment was verified by direct sequencing. The construct was then expressed in *Escherichia coli* BL21(DE3) strain, whose genome carries the RNA polymerase T7 gene under the control of a *lac* UV5 promoter. Gene expression was induced by isopropyl-β-d-thiogalactopyranoside (IPTG); however, the recombinant protein was not expressed in large amounts, even after long induction times (3 h); concentration was < 5 mg l^−1^. Possibly, overproduction was precluded because the archaeal factor exerted some toxic effect on *E. coli* cells; however, the amount of expressed protein was sufficient for subsequent purification.

**Fig. 1:**
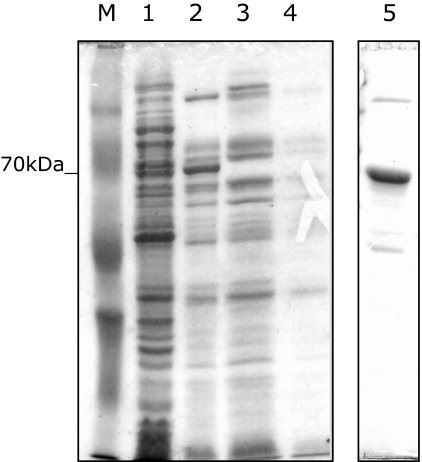
Purification of recombinant aIF2/5B. Proteins were analysed on a 12.5% SDS-PAGE stained with Coomassie brilliant blue. Lanes: M, molecular weight marker; 1, total extract from *E. coli* BL21 transformed with pRSETB-His6-aIF2/5B; 2, same extract as in 1 heated at 70°C for 10 min and centrifuged; 3, 4, flow-through proteins from the affinity column; 5, purified aIF2/5B eluted from the chromatographic column.

The aIF2/5B protein was purified from *E. coli* extracts by a two-step method: a differential thermal denaturation followed by affinity chromatography. Results in Fig. 1 show that heat treatment (70°C) of transformed *E. coli* extracts eliminates most proteins of the host, considerably enriching the lysates with the thermostable archaeal polypeptide. Removal of precipitated proteins by centrifugation resulted in a 10-fold enrichment in the aIF2/5B His-tagged protein (about 70 kDa on SDS-PAGE 12.5%). Final purification of the thermostable factor was accomplished by affinity chromatography of the heat-treated cell extracts on a Ni^+^ agarose column. An aliquot of the purified recombinant aIF2/5B was used to generate polyclonal antibodies in mouse, which allowed to study the localization of the protein in *S. solfataricus* cell extracts.

### Construction of ‘chimeric’ bacterial–archaeal IF2/5B factors

**Fig. 2:**
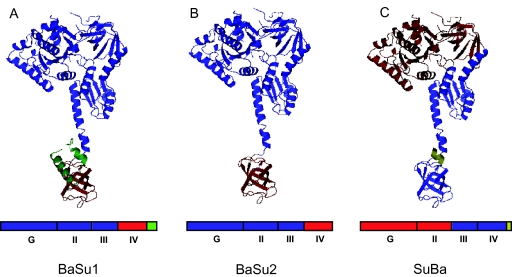
Schematic representation of the three IF2 chimeric proteins. (A) BaSu1; (B) BaSu2; (C) SuBa. On the three-dimensional structure of *Methanobacterium thermoautotropicum* IF2/5B (PDB ID 1G7R; Roll-Mecak *et al*., 2000) the different domains have been indicated with the following colour code: blue, domains from *B. stearothermophilus* IF2; red, domains from *S. solfataricus* aIF2/5B; green, C-terminal α-helices (H13 and H14) from *S. solfataricus*; gold, C-terminal amino acids from *B. stearothermophilus* IF2. A schematic representation of the primary structure showing the different domains in the same colour code is presented below.

To investigate the degree of functional similarity existing between the archaeal and the bacterial IF2-like factors, we also constructed ‘chimeric’ proteins by swapping domains between *S. solfataricus* aIF2/5B and*B. stearothermophilus* IF2. As shown by crystallographic studies (Roll-Mecak *et al*., 2000), the IF2-like proteins consist of four principal domains, some of which can be further divided in subdomains (Gualerzi *et al*., 1991; Spurio *et al*., 2000). The various IF2 domains are known to carry out different tasks. Domain I (or G) contains the guanine nucleotide binding centre and interacts with the GTPase centre of the 50S subunit (Allen *et al*., 2005). Domains II and III seem to be involved in the interaction with 30S and 50S ribosomes, respectively; domain II also makes contact with IF1 (Allen *et al*., 2005). Domain IV interacts with the large ribosomal subunit (Marzi *et al*., 2003; Allen *et al*., 2005) and, in the bacterial factor, contains the fMet-tRNAi binding site (Guenneugues *et al*., 2000). The hybrid proteins constructed for this study are shown in Fig. 2. The construct termed BaSu1 included the N-terminal domains G, II and III of bacterial IF2 and the C-terminal domain IV from *S. solfataricus* aIF2/5B. The construct BaSu2 was similar to BaSu1 except that *S. solfataricus* domain IV missed the two C-terminal α-helices characteristic of the archaeal proteins and not found in the bacterial one. Finally, the chimera called SuBa included domains G and II from the archaeal factor and domains III and IV from the bacterial one. This latter protein was poorly soluble and therefore could not be used in the full range of functional tests as the other two (see below).

The recombinant ‘chimeric’ proteins were expressed in *E. coli* and purified as described in *Experimental procedures.*

### aIF2/5B interacts with ribosomes during translational initiation

That aIF2/5B functions as a translation initiation factor was first indicated by experiments where the protein from *M. jannaschii* could partially substitute *in vivo* for the function of eIF5B, its eukaryal homologue (Lee *et al*., 1999). To verify these findings, and to obtain a more detailed insight about the role of the protein, we began with determining the abundance of aIF2/5B and its cellular localization in various experimental conditions.

Preliminarily, we studied the presence of the protein in batch-fractionated *S. solfataricus* cellular extracts: a whole-cell lysate (S-30), a post-ribosomal supernatant (S-100) and a high-salt ribosome wash (HSRW), prepared by treating *S. solfataricus* ribosomes with high concentrations of salt (NH_4_Cl 2 M). Such treatment removes any proteins that are loosely associated with the ribosomes, mainly a pool of translation factors. The various protein preparations were fractionated by SDS-PAGE and subjected to Western blotting with the specific anti-aIF2/5B antibodies.

**Fig. 3:**
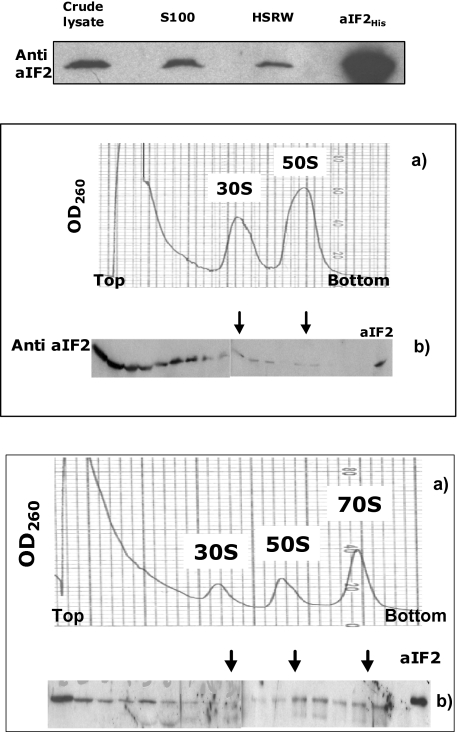
Localization of aIF2/5B in cell lysates Top: Western blot analysis with anti-aIF2/5B antibodies of batch-fractionated cell extracts. S-100: post-ribosomal supernatant; HSRW: high-salt ribosome wash; aIF2_His,_ recombinant aIF2/5B. Middle: (a) Density gradient fractionation of a *S. solfataricus* cell lysate, showing the position of the 30 and 50S ribosomes. (b) Western blot analysis of the gradient fractions with anti-aIF2/5B antibodies. The arrows indicate the position of the fractions containing the ribosomal subunits. The last lane is the control with the purified recombinant protein. Bottom: (a) gradient fractionation of a *S. solfataricus* cell lysate programmed for translation following incubation at 70°C for 3 min. The position of the 30S, 50S and 70S ribosomes is shown. (b) Western blot analysis of the gradient fractions with anti-aIF2/5B antibodies. The arrows indicate the position of the fractions containing the ribosomal subunits. The last lane is the control with the purified recombinant protein.

As shown in Fig. 3 (top), the aIF2/5B antiserum (but not the pre-immune control, data not shown) recognized in all preparations a single polypeptide, whose size (about 67 kDa) was that expected for the endogenous aIF2/5B. This result confirmed that the gene for aIF2/5B is actively translated in *S. solfataricus*; the protein is abundant in the cytoplasmic fraction (S-100), and also present in lesser amount in the ‘ribosome wash’.

More detailed information was obtained by fractionating a sample of *S. solfataricus* whole-cell lysate (S-30) on sucrose density gradient. The individual fractions were assayed for the presence of aIF2/5B by Western blotting with the specific antibodies. As shown in Fig. 3 (middle), the gradient optical density (260 nm) profile revealed the presence of two main peaks corresponding to the 30S and 50S ribosomal subunits. No peak at 70S was detected, indicating that no stable monomeric ribosomes are present in the ‘resting’ cell lysates; indeed, it is known that the 70S ribosomes of *Sulfolobus* and other Crenarchaea readily dissociate into subunits unless engaged in mRNA translation (Londei *et al*., 1986). Western blotting showed that most of aIF2/5B localized in the top fractions with low-molecular-weight material, while a smaller amount was visible in the fractions containing the 30S and 50S ribosomal subunits.

The same experiment was then carried out on lysates programmed for translation (Condo *et al*., 1999) and incubated for a short time (2 min) at 70°C. The results, illustrated in Fig. 3 (bottom), show that a peak of 70S monomers now appeared, while an appreciable amount of aIF2/5B moved from the low-molecular-weight fractions to localize on the ribosomes. Significantly, a fraction of the protein associated with the 70S peak, while the amount of aIF2/5B bound to the subunits, especially the 50S ones, was also increased. On the other hand, when the samples were incubated for longer periods of time (45 min), most of aIF2/5B was again found in the low-molecular-weight fractions (results not shown), indicating that after the initial phases of translation it fell off the 70S ribosomes, most of which were now engaged in the elongation cycle. These results showed that aIF2/5B interacted with the ribosomes during translational initiation and that, like its bacterial homologue, it bound to both ribosomal subunits and also to 70 ribosomes.

Next, we inquired whether purified recombinant aIF2/5B could interact with the archaeal ribosomes independently of other translational components. To this end, purified *S. solfataricus* ribosomes were incubated with recombinant aIF2/5B for 10 min at 70°C, and the samples were fractionated on density gradients as described above. The fractions were subjected to Western blotting with anti-His antibodies, to detect exclusively the recombinant protein. The latter was found to interact with both ribosomal subunits with a low affinity, as observed for the native factor in the ‘resting’ cell lysates (results not shown). Therefore the archaeal factor, like the bacterial one, could interact by itself with both ribosomal subunits in the absence of other factors or of tRNAi.

### Conservation of the ribosome binding sites in archaeal and bacterial IF2 proteins

It is known that the principal ribosome-interacting domains of bacterial IF2 are located in the ‘cup’ of the chalice. Specifically, the N-terminal domain and domain II contact the 30S subunit while domain III (and also in part domain IV) interacts with the 50S subunit (Marzi *et al*., 2003; Allen *et al*., 2005; Caserta *et al*., 2006). The evolutionary conservation of the ribosomal binding sites for the IF2-like proteins in bacteria and archaea was explored by determining the capacity of IF2 and aIF2/5B to interact with the heterologous ribosomes, and by assaying the ribosome-binding capacity of ‘chimeric’ factors, obtained by ‘domain swapping’ between *S. solfataricus* aIF2/5B and IF2 of the thermophilic bacterium *B. stearothermophilus* (see Fig. 2).

**Fig. 4:**
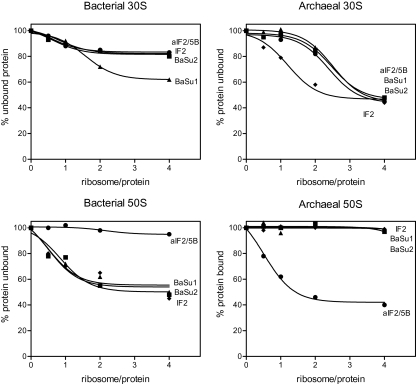
Interaction of native and chimeric IF2 factors with archaeal and bacterial ribosomal subunits. The ribosome-bound fraction of the various proteins was determined as described in *Experimental procedures*. Circle: recombinant *S. solfataricus* aIF2/5B; diamond: recombinant *B. stearothermophilus* IF2; triangle: BaSu1 chimera; square: BaSu2 chimera.

As shown in Fig. 4 (top), both recombinant aIF2/5B and IF2 bound with low affinity to 30S subunits regardless of their source. Accordingly, the chimeric factors BaSu1 and BaSu2, containing the bacterial ribosomal binding domains, interacted with small subunits from either source.

Binding to the 50S subunits, in contrast, appeared to be restricted within the homologous domain (Fig. 4 bottom). Neither aIF2/5B nor IF2 interacted with the heterologous large subunits. As regards the chimeric proteins, BaSu1 and BaSu2 bound to *B. stearothermophilus* large subunits with an affinity comparable to that of the intact bacterial protein, while being unable to recognize the archaeal particles. Unfortunately, the hybrid protein SuBa, endowed with the archaeal ribosome-binding domains, could not be tested in this assay because of its poor solubility, which led to its appearance in the precipitate after centrifugation even in the absence of ribosomes.

### GTPase activity

**Fig. 5:**
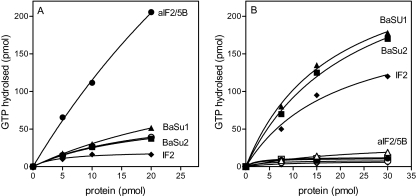
GTPase activity of the native and chimeric IF2 factors A. GTPase activity of the native and chimeric IF2 factors in the presence of *S. solfataricus* 70S ribosomes. B. GTPase activity of the native and chimeric IF2 factors in the presence of *B. stearothermophilus* 70S ribosomes. Circle: recombinant *S. solfataricus* aIF2/5B; diamond: recombinant *B. stearothermophilus* IF2; triangle: BaSu1 chimera; square: BaSu2 chimera. Open symbols refer to determinations made in the absence of ribosomes.

The data on the ribosome-binding capacity of IF2-like proteins were confirmed and extended by performing GTPase assays on the native and chimeric proteins, with both bacterial and archaeal ribosomes (Fig. 5). First, we determined that aIF2/5B has indeed a ribosome-dependent GTPase activity, which was only observed at high temperature (above 60°C) and required only the presence of the recombinant factor and purified *S. solfataricus* ribosomes (Fig. 5A).

As expected from the ribosome binding data, bacterial IF2 was unable to hydrolyse GTP in the presence of the archaeal ribosomes, and the same was true for both BaSu1 and BaSu2 hybrid factors (Fig. 5A). Conversely, *B. stearothermophilus* ribosomes were unable to trigger the GTPase activity of aIF2/5B, but did trigger that of the BaSu1 and BaSu2 chimerae (Fig. 5B). Unexpectedly, the protein SuBa, composed of the archaeal domains G and II and of bacterial domains III and IV (Fig. 2), had a ribosome-independent GTPase activity (not shown). The reason for this behaviour is unclear, but could conceivably be ascribed to some folding defect of the G domain in the hybrid factor.

### Interaction of IF2 proteins with initiator tRNA

The next issue we explored was the interaction of aIF2/5B with initiator tRNA. It is known that bacterial IF2 binds fMet-tRNAi in solution (Guenneugues *et al*., 2000; Krafft *et al*., 2000; Spurio *et al*., 2000). Recently, aIF2/5B from the archaeon *Pyrococcus abyssi* was also reported to interact in solution with bacterial Met- and fMet-tRNAi (Guillon *et al*., 2005).

However, eukaryal IF5B does not bind tRNAi by itself and probably intervenes only indirectly in tRNAi/ribosome interaction (Pestova *et al*., 2000). It should be noted that aIF2/5B and eIF5B are closer in sequence to each other than either is to IF2 and both lack the critical amino acids in the C-terminal domain which have been shown to be essential for fMet-tRNAi binding (Guenneugues *et al*., 2000). On the score of these data, we deemed it important to analyse the interaction of *S. solfataricus* aIF2/5B with initiator tRNA.

To this end, we determined whether, and to which extent, the native archaeal and bacterial factors and the hybrid proteins were able to prevent the loss of the amino acid from Met-tRNAi (archaeal) or from fMet- and Met-tRNAi (bacterial). This technique is based on the fact that the interaction between tRNAi and the IF2 factor in solution shields from hydrolysis the ester bond anchoring the methionine to the tRNA.

**Fig. 6:**
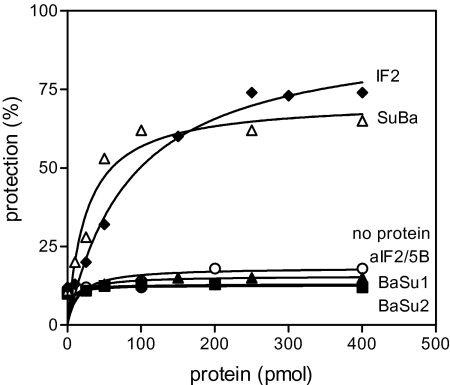
Protection of fMet-tRNAi from alkaline hydrolysis in the presence of native and chimeric IF2 proteins. The extent of fMet-tRNAi protection was determined as described in *Experimental procedures*. Closed circle: recombinant *S. solfataricus* aIF2/5B; closed diamond; recombinant *B. stearothermophilus* IF2; closed triangle: BaSu1 chimera; closed square: BaSu2 chimera; open triangle: SuBa chimera; open circle: no proteins added.

Unformylated Met-tRNAi of either bacterial or archaeal origin was not protected by any of the factors assayed (not shown). Instead, as illustrated in Fig. 6, fMet-tRNAi (bacterial) was strongly protected by both *B. stearothermophilus* IF2 and the chimeric protein SuBa, containing the C-terminal domains III and IV of bacterial origin. In contrast, no protection was exerted by aIF2/5B and by the chimerae BaSu1 and BaSu2, containing an archaeal domain IV in a bacterial context.

On the whole, the results demonstrated that the domain IV of the archaeal and bacterial proteins behaves differently with respect to initiator tRNA binding. Archaeal domain IV is unable to interact stably in solution with either Met- or fMet-tRNAi, both bacterial and archaeal, while bacterial domain IV binds fMet-tRNAi with high affinity but interacts poorly, if at all, with unformylated Met-tRNA. Moreover, it is evident from the data that tRNAi binding is a property of bacterial domain IV itself, independent of the rest of the protein, as already shown by Spurio *et al*. (2000).

### Overexpression of aIF2/5B enhances translation

**Fig. 7:**
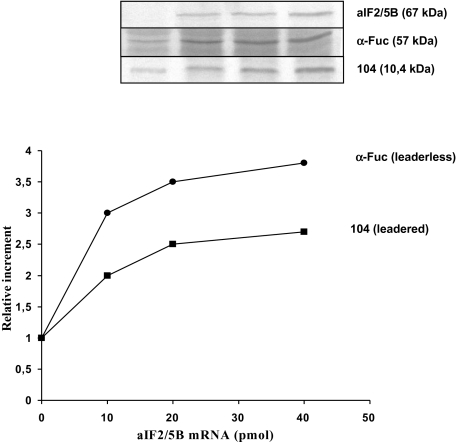
aIF2/5B enhances *in vitro* translation of leadered and leaderless mRNAs Top. Autoradiography of SDS-PAGEs after electrophoresis of the radiolabelled proteins synthesized in a *S. solfataricus in vitro* translation systems programmed with increasing amounts of aIF2/5B mRNA and fixed amounts of a leaderless (α-fucosidase) or a leadered (104) mRNA. Lane 1: 10 pmol of α-fuc or 10 pmol of 104 mRNA in the absence of aIF2/5B mRNA; Lanes 2–4: same with 10, 20 and 40 pmol, respectively, of aIF2/5B mRNA. Bottom. Curve obtained by quantifying with a Molecular Dynamics Phosphoimager the radioactivity retained in the bands as shown in the top panel. The relative increment on the *y*-axis is the ratio between radioactivity values in the absence of added aIF2/5B mRNA and values measured in the presence of the indicated amounts of aIF2/5B mRNA.

The next experiments were aimed at investigating the function of aIF2/5B in translation. To this end, we began with determining whether the presence of the factor in excess amounts exerted any effect on the efficiency of *in vitro* protein synthesis. These experiments could not be carried out by simply adding increasing amounts of the recombinant factor to samples programmed for *in vitro* translation, as the storage buffer in which recombinant aIF2/5B was kept stable inhibited unspecifically the *S. solfataricus in vitro* translation system. To circumvent this problem, we obtained another clone of the aIF2/5B gene, including a tract of the 5′ region upstream of the AUG codon, which contained the putative ribosome binding site. The construct included a T7 polymerase promoter which was used to obtain an *in vitro* run-off transcript that was translated in the cell-free system yielding a product of the expected size of aIF2/5B (Fig. 7, top). This mRNA was then added in increasing amounts to cell-free systems also programmed with a fixed amount of a reporter mRNA, encoding an housekeeping protein (Condo *et al*., 1999). Two different reporter mRNAs were employed: one, encoding a ribosomal protein, was leadered and endowed with a strong SD motif ahead of the AUG initiation codon. Another, encoding an enzyme (α-fucosidase), had a mini-5′ UTR of only nine nucleotides and lacked any SD motif (Cobucci-Ponzano *et al*., 2006).

As shown in Fig. 7, the *in vitro* synthesis of both reporter proteins, especially that encoded by the leaderless mRNA, was significantly enhanced in a fashion proportional to the amount of aIF2/5B mRNA added to the system. To rule out the possibility that this effect was due to an unspecific stimulation of protein synthesis exerted by added RNA rather than by aIF2/5B itself, control experiments were carried out where the amount of one reporter mRNA was increased while keeping constant that of aIF2/5B mRNA. This procedure, however, failed to stimulate aIF2/5B translation, showing that RNA does not enhance translation unspecifically (results not shown). On the whole, the results in Fig. 7 indicate that aIF2/5B plays an important role in both the ‘leadered’ and ‘leaderless’ pathways for translation initiation, but especially in the latter one.

### aIF2/5B stimulates met-tRNAi binding to ribosomes

The results illustrated in the previous paragraph suggested that aIF2/5B acted at some crucial step common to both leadered and leaderless initiation. Previous literature data on the yeast IF2-homologous factor (eIF5B), the closest homologue of the archaeal protein, indicated that it enhanced the interaction of Met-tRNAi with the ribosome (Choi *et al*., 1998), suggesting that this could be the function common to all IF2-like proteins. However, eIF5B is apparently non-essential in yeast (albeit very important for a normal growth), in agreement with the fact that the Met-tRNAi binding factor is a different protein (eIF2) in eukaryotes. The same is true for archaea, where the Met-tRNAi-binding protein is a trimeric factor homologous to eIF2 (Yatime *et al*., 2004; Pedulla *et al*., 2005).

The archaeal homologue of eIF2 (a/eIF2), similar to aIF2/5B, binds *in vitro* to both ribosomal subunits; however, ribosome binding of aIF2/5B and of a/eIF2 is mutually exclusive, demonstrating that the factors occupy similar positions on the ribosomes (P. Londei, unpubl. results). Given the ability of aIF2/5B to bind the ribosomes in the absence of other factors, we asked whether it was also able to stimulate to some extent tRNA/ribosome interaction. To this end, *S. solfataricus* 70S ribosomes were incubated with purified, [^35^S]-labelled, *S. solfataricus* Met-tRNAi and increasing amounts of aIF2/5B in the presence of GTP. The ribosomes had been previously treated with high-salt (2 M NH_4_Cl) to remove any bound extrinsic factors; the absence of aIF2/5B was verified by Western blotting (not shown).

**Fig. 8:**
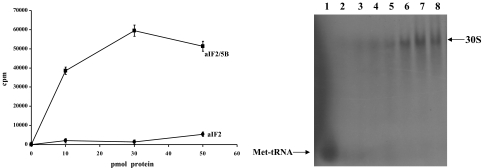
aIF2/5B stimulates the interaction of met-tRNAi with the archaeal 30S subunits. The amount of ribosome-bound met-tRNAi was determined by electrophoresis of the incubation mixtures on non-denaturing gels (see *Experimental procedures*). The autoradiography of the original gel is shown on the right. Lane 1: *S. solfataricus*[^35^S]Met-tRNAi without ribosomes; lane 2: *S. solfataricus*[^35^S]Met-tRNAi and *S. solfataricus* 70S ribosomes without factors; lanes 3–5: *S. solfataricus*[^35^S]Met-tRNAi and *S. solfataricus* 70S ribosomes with increasing amounts (10, 30, 50 pmol) of *B. stearothermophilus* IF2; lanes 6–8: *S. solfataricus*[^35^S]Met-tRNAi and *S. solfataricus* 70S ribosomes with increasing amounts (10, 30, 50 pmol) of *S. solfataricus* aIF2/5B. The curve on the left is obtained by quantifying with a Molecular Dynamics Phosphoimager the radioactivity retained in the bands as shown in the panel on the right. Squares: increasing amounts of aIF2/5B; circles: Increasing amounts of bacterial IF2.

tRNA/ribosome binding was monitored by electrophoresing the incubation mixtures on non-denaturing gels, which allow separation and detection of ribosomes and ribosome–tRNA complexes (Acker *et al*., 2006). The results, shown in Fig. 8, revealed that the addition of aIF2/5B to the incubation mixture stimulated appreciably the binding of Met-tRNAi to the ribosomes, specifically to the 30S ribosomal subunits. As expected, bacterial IF2 was unable to stimulate Met-tRNAi interaction with *S. solfataricus* ribosomes.

We concluded that aIF2/5B could promote Met-tRNAi/ribosome interaction even in the absence of the specific Met-tRNAi binding factor a/eIF2, and also in the absence of a direct interaction in solution between aIF2/5B and Met-tRNAi.

**Fig. 9:**
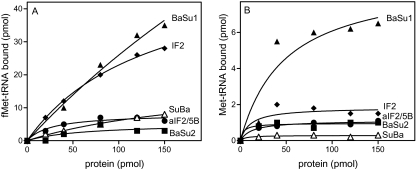
Interaction of fMet- and met-tRNAi with bacterial ribosomes in the presence of native and chimeric IF2 factors A. Stimulation of fMet-tRNAi binding to bacterial 30S subunits by increasing amounts, as indicated, of native and chimeric IF2 factors. B. Stimulation of Met-tRNAi binding to bacterial 30S subunits by increasing amounts, as indicated, of native and chimeric IF2 factors. Closed circle: recombinant *S. solfataricus* aIF2/5B; closed diamond; recombinant *B. stearothermophilus* IF2; closed triangle: BaSu1 chimera; closed square: BaSu2 chimera; open triangle: SuBa chimera.

These results were extended and corroborated by assaying the ability of the native and chimeric IF2-like factors to stimulate the binding of both Met-tRNAi and fMet-tRNAi to bacterial ribosomes. As shown in Fig. 9, the interaction of fMet-tRNAi with *B. stearothermophilus* 30S subunits was stimulated, as expected, by bacterial IF2, but also to a comparable extent by the chimeric protein BaSu1, containing the complete domain IV of archaeal origin which, as seen above, is unable to interact in solution with both fMet- and Met-tRNAi. Interestingly enough, however, the protein BaSu2, which has the same ribosome-binding pattern as BaSu1 (Fig. 4) but lacks a C-terminal fragment including the archaeal-specific C-terminal α-helix, was totally inactive in promoting ribosomal binding of either Met- or fMet-tRNAi. These results suggest that the C-terminal segment of archaeal domain IV does interact with tRNAi on the ribosomal surface, and that it is essential in properly adjusting the tRNA in its ribosomal binding site. In agreement with this surmise, BaSu1 also stimulated the binding of Met-tRNAi to bacterial ribosomes, although to a much lesser extent than that of fMet-tRNAi. In contrast, ribosomal binding of Met-tRNAi was not promoted by IF2, in agreement with the observation that the bacterial domain IV recognizes specifically the formyl group on the initiator amino acid.

## Discussion

The IF2-like proteins (IF2 in bacteria, eIF5B in eukarya, aIF2/5B in archaea) belong to the small group of translation initiation factors that are represented in all three domains of life. Because of its universal conservation, the IF2-like factor must have been present in the last universal common ancestor (LUCA) of extant cells and therefore must have exerted some essential function already at that ancestral stage of cellular evolution. However, modern IF2-like proteins from bacteria and eukarya have incurred a substantial functional divergence, making it important to study the archaeal homologue to reach some understanding about their shared role in translation initiation.

We have shown in this study that *S. solfataricus* aIF2/5B is loaded on the ribosomes during translational initiation and promotes the binding of initiator tRNA (Met-tRNAi for the archaea) to the ribosomal P site, thereby stimulating *in vitro* translation of both leadered and leaderless mRNAs. Unlike bacterial IF2, but like eukaryal eIF5B, *S. solfataricus* aIF2/5B does not interact directly with tRNAi prior to binding to the ribosome. However, archaeal aIF2/5B may well be somewhat heterogeneous in their tRNAi binding properties, as recombinant aIF2/5B from the hyperthermophilic euryarchaeon *P. abyssi* was found to protect fMet-tRNAi from alkaline deacylation in solution (Guillon *et al*., 2005). In any event, it is known that in archaea, as in eukarya, the Met-tRNAi-binding protein is another factor, the trimeric protein a/eIF2 (Pedulla *et al*., 2005). In summary, aIF2/5B appears to have features generally closer to those of its eukaryal homologue, albeit still sharing traits with the bacterial factor, such as the capacity of autonomous interaction with both ribosomal subunits and a limited capacity, only in some archaeal species, of interacting directly with tRNAi.

The degree of evolutionary divergence incurred by the bacterial and the archaeal IF2-like factors has been analysed in some detail in this work by producing ‘chimeric’ factors, i.e. hybrid proteins obtained by swapping domains between *S. solfataricus* aIF2/5B protein and IF2 of the thermophilic bacterium *B. stearothermophilus* (Fig. 2). We have examined a number of properties, such as ribosome binding, GTP hydrolysis, tRNAi binding and stimulation of tRNAi/ribosome interaction.

As regards the mode of interaction with the ribosomes, a couple of recent works (Marzi *et al*., 2003; Allen *et al*., 2005) have revealed that IF2 (bacterial) is sandwiched between the two ribosomal subunits, contacting the 30S particle mainly by domain II and the 50S one by domains III and IV. We have found here that all of the IF2-like proteins, both native and chimeric, can interact with either bacterial and archaeal small ribosomal subunits, thereby indicating that domain II of IF2-like factors has maintained a broad ribosome-binding capacity. Interestingly, this is not true for the domains (III and IV) involved in the interaction with the 50S subunit, which can only recognize the homologous ribosomes. Thus, both hybrid factors BaSu1 and BaSu2, having the bacterial domains G, II and III and archaeal domain IV, interact with bacterial 50S with the same affinity as the native bacterial IF2, while native aIF2/5B does not bind at all to bacterial 50S ribosomes. Accordingly, activation of the ribosome-dependent GTPase activity of the IF2 factors depends on their ability to interact with the large ribosomal subunit. These results suggest that the interaction of IF2-like protein with the 50S subunit is the one having the highest degree of specificity and is probably the most important for the proper setting of the translation initiation complex. Indeed, recent data indicate that 30S-bound bacterial IF2 rearranges its position when the large subunit joins the pre-initiation complex (Marzi *et al*., 2003).

The experiments with the chimeric proteins also highlight the details of IF2 interaction with initiator tRNA, confirming that the domains IV of *B. stearothermophilus* and *S. solfataricus* factors have clearly different tRNAi-binding capacities. The presence of the bacterial domain IV is necessary and sufficient to allow binding of fMet-tRNAi, as shown by the behaviour of the hybrid factor SuBa. On the other hand, archaeal domain IV remains unable to interact with either Met- or fMet-tRNAi even when placed in a bacterial context. These results also indicate that, as previously shown for *B. stearothermophilus* IF2 (Spurio *et al*., 2000), the bacterial domain IV retains a full tRNAi-binding capacity even in isolation or when inserted in a largely heterologous context. However, a salient result of the present work is that a direct interaction in solution between the IF2-like factor and tRNAi is not required to promote the ribosomal binding of the latter. This is shown by the behaviour of the chimeric protein SuBa1, which cannot interact directly with fMet-tRNAi but can still stimulate its binding to bacterial 30S subunits, even more efficiently than the native bacterial IF2. However, a proper contact between tRNAi and domain IV of IF2 on the ribosomal surface is probably important for the correct adjustment of tRNAi in the ribosomal P site during the formation of the 70S complex. This is shown by the behaviour of the hybrid factor SuBa2, which, unlike SuBa1, is inactive in promoting the binding of fMet-tRNAi to the bacterial 30S subunits. The two chimeric proteins differ only for the absence, in SuBa2, of a C-terminal tract forming two α-helical structures (see Fig. 2). Possibly, this prevents a correct interaction between tRNAi and IF2, either because domain IV is not correctly folded or because the missing α-helices have a direct role in contacting the tRNAi.

Upon the whole, the available data suggest that, despite their evolutionary divergence, the IF2-like factors are in all cases required for a correct entry of the tRNAi in the ribosomal P site, regardless of whether the proper tRNAi is chosen by IF2 itself (as in bacteria) or by another factor (as in archaea and eukarya). The enhancement of tRNAi/ribosome interaction by IF2 may also in part be due to the fact that the factor promotes the formation of 70S or 80S ribosomes from the separate subunits. Also, it must be mentioned that all IF2-like proteins seem to interact, either in solution or on the ribosome, with the IF1-like factor, another universal protein (Roll-Mecak *et al*., 2001; Marintchev *et al*., 2003; Londei, 2005). IF1 (IF1A in the archae/eukarya) functions as a blocker of the A site and, at the same time, improves the affinity of IF2 for the ribosome, helping the proper placement of tRNAi on the translation initiation region of the mRNA.

It is interesting to point out that bacterial IF2 binds fMet-tRNAi by recognizing mainly the formyl group on the amino acid, as described earlier (Guenneugues *et al*., 2000) and also shown here by the fact that unformylated Met-tRNAi is essentially inactive in the assays carried out with bacterial IF2. This suggests that the formylation of the N-group of the tRNAi-bound methionine is a feature specifically evolved in the bacterial lineage that has allowed the latter to use a single factor for recognizing a specific initiator tRNA and properly adjusting it on the ribosome. In the archaea and in the eukarya, the same functions require two proteins, one (a/eIF2) to bind Met-tRNAi and another (a/eIF2/5B) to adapt it in the P site. In all kingdoms, the IF2-like factor also seems to promote the joining of the ribosomal subunit to form a monomeric 70S or 80S ribosome.

Why the bacteria and the archaea/eukarya have diverged in their usage of tRNAi binding factors must remain uncertain for the time being. The IF2-like factors belong to a family of translational GTPases that includes the elongation factors 1 A (Tu in bacteria) and 2 (G in bacteria) as well as the gamma subunit of the a/eIF2 trimer (Keeling *et al*., 1998). If the ancestral IF2 protein resembled the modern archaeal/eukaryal factor in lacking the capacity to interact with a particular tRNA, a possible scenario for translational initiation at the LUCA stage would be that any aminoacyl-tRNA could have functioned as initiator, provided that the IF1/IF2 complex was there to guide it in the ribosomal P site. The later divergence of the initiation pathway in the different primary domains entailed the evolution, by the bacterial IF2, of the ability to recognize specifically a Met-tRNA carrying a formylated amino acid. The archaea/eukarya recruited another tRNA-binding GTPase, the gamma subunit of a/eIF2, to interact with Met-tRNA (Londei, 2005), while retaining the IF2-like factor to stabilize the tRNAi in the P site and facilitate the joining of the ribosomal subunits.

## Experimental procedures

### Preparation of *S. solfataricus* cellular extracts and other cellular fractions

*Sulfolobus solfataricus* cells were disrupted by grinding with twice their weight of alumina powder (Alcoa) while gradually adding 2.5 ml per gram wet weight of cells of ribosome extraction buffer [20 mM Tris-HCl pH 7.0, 10 mM Mg(OAc)_2_, 40 mM NH_4_Cl, 3 mM DTT, 2.5 μg ml^−1^ RNase-free DNase]. Tris-HCl buffer was replaced by 20 mM triethanolamine/HCl (pH 7.4) when fixation with 6% HCHO to stabilize 70S ribosomes was required. Alumina and cellular debris were removed by centrifuging twice at 30 000 *g* for 30 min. The clarified supernatant obtained (S-30) was stored at −80°C.

Crude lysates (S30) were centrifuged in a Spinco Ti 50 rotor at 45 000 rev. min^−1^ for 2 h (4°C) to separate the ribosomes from a supernatant (S-100) containing bulk tRNA and cytoplasmic proteins.

Crude total tRNA was obtained by extracting the post-ribosomal supernatants three times with phenol and by precipitating the last aqueous phase with 2.5 volumes of 95% ethanol.

The pellet of ribosomes (termed crude ribosomes) was further purified by re-suspending in buffer [20 mM Tris-HCl pH 7.0, 10 mM NH_4_Cl, 500 mM Mg(OAc)_2_, 2 mM DTT] followed by centrifugation in a Spinco Ti 50 rotor at 45 000 rev. min^−1^ for 5 h (4°C).

Initiation factor-free ribosomes were obtained by re-suspending the ribosome pellets in a high-salt buffer [20 mM Tris-HCl, pH 7.0, 2 M NH_4_Cl, 10 mM Mg(OAc)_2_, 2.0 mM dithiothreitol]. The ribosome suspension was layered onto a 7.0 ml cushion of 0.5 M sucrose made in high-salt buffer and centrifuged in a Spinco Ti 50 rotor operated at 45 000 r.p.m. for 3 h (4°C). The ribosome pellets (termed ‘salt-washed ribosomes’) were lastly re-suspended at A_260_ = 500 in ribosome extraction buffer containing 10% (v/v) glycerol.

### Purification of ribosomal subunits

To separate the ribosomal subunits, salt-washed ribosomes were re-suspended in 20 mM Tris-HCl, pH 7.0, 500 mM NH_4_Cl, 10 mM Mg(OAc)_2_, 2.0 mM dithiothreitol. Aliquots (1 ml) of the ribosome suspensions (= 40 A_260_ units) were layered onto 38 ml linear, 10–30% (w/v) sucrose density gradients made in the ribosome-suspending buffer. The gradients were centrifuged in a Beckman SW 27 rotor operated at 18 000 rev. min^−1^ and 5°C for 18 h. Fractions corresponding to the 30S and 50S peaks of A_260_ were separately pooled and the particles therein were precipitated by the addition of 2 volumes of ethanol. After low-speed centrifugation the subunit pellets were re-suspended in ribosome extraction buffer containing 10% (v/v) glycerol and stored at −20°C.

Ribosomal subunits from *B. stearothermophilus* 799 strain were prepared essentially as described (Ohsawa and Gualerzi, 1983).

### Preparation of *S. solfataricus* native $tRNA_{i}^{Met}$

Purified native initiator met-tRNAi was obtained by affinity purification as follows. A 30-mer oligonucleotide (5′-GCTTCAGGGACCAAGTTTAGGTCCGGGGCG biotine-3′) was synthesized, complementary to the 3′ moiety of the sequence of *S. solfataricus* initiator met-tRNAi as deduced from the published genome sequence (She *et al*., 2001).

The biotinylated oligo (10 μg) was mixed with 0.6 mg of streptavidin MagneSphere paramagnetic particles (Promega), and the mixture was incubated at room temperature for 30 min in 500 μl of 10 mM Tris-HCl, pH 7.5. The particles were washed three times with 600 μl of 10 mM Tris-HCl, pH 7.5, then three times with 600 μl of 6× SSC (900 mM NaCl, 90 mM sodium citrate). The crude total tRNA was diluted to a final optical density of 100 A_260_ unit ml^−1^ with 6× SSC; 100 μl of this solution was mixed with 0.6 mg particles coupled with the oligo. The sample was incubated at room temperature for 30 min with continuous mixing; the particles were then separated from the supernatant using the appropriate magnetic separator and washed several times with 1 ml of 3× SSC until the A_260_ was close to zero. The particles were finally re-suspended in 100 μl of 0.1× SSC and incubated at 60°C for 2 min, after which the particles were magnetically separated from the supernatant which was saved. The whole operation was repeated once more. Purified tRNA was finally recovered from the pooled supernatants by precipitation with 2.5 volumes of 95% ethanol containing 500 mM NH_4_(OAc) and 0.2 mg ml^−1^ (final concentration) glycogen. The pellet was re-suspended in a suitable volume of sterile H_2_O.

### Charging of $tRNA_{i}^{Met}$

Native *S. solfataricus* $\text{t}\text{R}\text{N}{\text{A}}_{\text{i}}^{\text{M}\text{e}\text{t}}$ in total tRNA, or purified native $\text{t}\text{R}\text{N}{\text{A}}_{\text{i}}^{\text{M}\text{e}\text{t}}$ (obtained as previously described), was charged with methionine using either a *S. solfataricus* S100 preparation freed of nucleic acids as the source of MetRT (Benelli *et al*., 2003) or a recombinant fragment of 547 amino acids of *E. coli* MetRT (courtesy of S. Blanquet and E. Schmitt, Ecole Politechnique, Palaiseau).

Aminoacylation of 4 μM $\text{t}\text{R}\text{N}{\text{A}}_{\text{i}}^{\text{M}\text{e}\text{t}}$ was carried out in the presence of 5 μl S-100, or 1.5 μM of *E. coli* MetRT, in 100 μl of reaction mixture containing 20 mM HEPES, pH 7.5, 100 μM Na_2_EDTA, 150 mM NH_4_Cl, 4 mM ATP, 10 mM MgCl_2_ and 0.1 mM unlabelled methionine or 10 μM [^35^S]-methionine (1000 Ci mM^−1^) respectively. If the bacterial enzyme was used, the reaction mixture was incubated for 30 min at 37°C; if the archaeal proteins were used, incubation was at 60°C for 15 min. At the end, the samples were phenol extracted, ethanol precipitated and re-suspended in 50 μl of 10 mM KCH_3_COOH, pH 5.5.

### Preparation of recombinant *S. solfataricus* aIF2/5B

The *S. solfataricus* aIF2/5B gene was amplified by PCR from genomic DNA using primers suitable for its insertion in the *E. coli* expression vector pRSETB. The construct, termed pRSETB-His_6_-aIF2/5B, was transformed in the *E. coli* strain BL21(DE3). Expression was induced at OD_600_ = 0.6 with 0.4 mM IPTG and the cells were grown for a further 4 h before harvesting. The cell pellet was re-suspended in Lysis buffer (50 mM NaH_2_PO_4_, 300 mM NaCl, 10 mM imidazole, pH 8.0) and incubated with lysozyme on ice for 30 min. The cells were lysed by sonication and the lysate was clarified by centrifugation at 5000 r.p.m. for 30 min at 4°C. The cleared lysate was incubated for 10 min at 70°C, and an aIF2/5B enriched lysate was obtained by centrifugation at 10 000 r.p.m. for 30 min. Thermostable His_6_-aIF2/5B was purified from the lysate incubating for 1 h on Ni-NTA agarose resin (Qiagen), washing with Wash buffer (50 mM NaH_2_PO_4_, 300 mM NaCl, 15 mM imidazole, pH 8.0) and eluting with Elution buffer (50 mM NaH_2_PO_4_, 300 mM NaCl, 250 mM imidazole, pH 8.0). The resulting 69.9 kDa His-tagged protein could be considered ∼95% pure. This preparation was concentrated with CENTRIPREP concentrators Millipore and dialysed against Storage buffer (20 mM TEA-HCl pH 7.1, 60 mM KCl). The concentration of the samples was determined by Bradford Assay. Aliquots of protein were stored at −80°C.

### Construction of ‘chimeric’ IF2 factors

#### 

##### BaSu1

Plasmid pRSETB containing *S. solfataricus aIF2/5B* gene was used as template for amplification of a DNA fragment encoding the C-terminal domain of the archaeal protein.

The forward primer corresponded to positions P468–P475 of *S. solfataricus* aIF2/5B amino acid sequence and was designed to insert a SphI restriction site. The reverse primer was complementary to a region downstream of the stop codon and inserted a BamHI restriction site.

The PCR product, after digestion with SphI and BamHI, was inserted into the pIM401 vector harbouring the *infB* gene of *B. stearothermophilus* (Brombach *et al*., 1986). The *infB* gene of the pIM401 plasmid had been previously manipulated by inserting a silent mutation which created a unique SphI restriction site at Gly 637 thus allowing removal of the bacterial C-terminal domain and subsequent insertion of the archaeal DNA fragment using the same restriction sites.

##### BaSu2

This chimera is essentially identical to BaSu1 except for the length of the archaeal C-terminal domain which is lacking its last 45 amino acids corresponding to the last two α-helices (H13, H14).

The forward primer was identical to the one used for BaSu1 while the reverse primer corresponded to positions D550–V556 of *S. solfataricus* aIF2/5B amino acid sequence and inserted a BamHI restriction site. A new TAA stop codon is inserted at position 557.

##### SuBa

This chimera comprises domains G and II of *S. solfataricus* aIF2/5B fused to the C domain of *B. stearothermophilus* IF2, including domains III and IV (see Fig. 2).

pRSETB plasmid containing *S. solfataricus aIF2/5B* gene was used as template for PCR amplification of the N-terminal portion of the chimera. Forward primer corresponded to the first amino acids of the recombinant *S. solfataricus* aIF2/5B and was designed to insert an EcoRI restriction site. The reverse primer corresponded to positions L325–P329 of *S. solfataricus* aIF2/5B and inserted a Xba1 restriction site. The PCR product, cleaved with EcoRI and XbaI, was inserted into the pXP401C vector harbouring the sequence of the C domain of *B. stearothermophilus* IF2 (L515-A741) (Gualerzi *et al*., 1991; Spurio *et al*., 2000). In all cases amplification was performed using Pfu DNA polymerase (Promega) under the conditions suggested by the supplier.

### Expression and purification of bacterial IF2 and of chimeric IF2 proteins

All recombinant plasmids were transferred into *E. coli* UT5600 carrying plasmid pcI857 (Elish *et al*., 1988). Induction of expression was as described (Spurio *et al*., 2000). Purification of *B. stearothermophilus* IF2, BaSu1 e BaSu2 was performed essentially as described (Gualerzi *et al*., 1991) with the exception that the chimeric proteins were eluted from the DEAE-cellulose (Whatman DE-52) using a linear gradient from 50 mM to 350 mM NH_4_Cl.

Overexpression of SuBa, under control of λ P_L_ promoter, was obtained as described for BaSu1 and BaSu2. However, as this chimera bears a histidine tail at its N-terminus, purification was performed essentially as described above for aIF2/5B, except that the heating step was omitted.

Purified chimeric proteins were dialysed against 20 mM Tris-HCl pH 7.1, 200 mM NH_4_Cl, 10% Glycerol and 1 mM DTT and then stored at −80°C.

Concentration of the proteins was determined by Bradford assay.

### Localization of endogenous aIF2/5B in a *Sulfolobus* cell lysate

Two reactions were prepared. The first contained 50 μl of *S. solfataricus* crude lysate (S-30) and was not incubated. The second contained 15 μl of S-30 extract and the components needed to activate translation *in vitro* (Condo *et al*., 1999): 10 mM KCl, 20 mM TEA-HCl pH 7, 10 mM Mg(OAc)_2_, 6 mM ATP, 2 mM GTP, 5 μg of bulk *S. solfataricus* tRNA, 20 μM methionine and 3 μg of *in vitro* transcribed mRNA coding for a *S. solfataricus* gene termed ORF104 (Condo *et al*., 1999). The samples were incubated at 73°C for 5 min, and the reaction was fixed on ice with 6% (final concentration) formaldehyde.

All the samples (50 μl each) were layered on 12 ml, linear, 10–30% sucrose gradients containing 10 mM KCl, 20 mM TEA-HCl (pH 7.5) and 10 mM MgCl_2_ which were centrifuged in a SW41 rotor at 4°C at 36 000 r.p.m. for 4 h. After centrifugation, the gradients were unloaded with an ISCO UA-5 gradient collector and 0.5 ml fractions were collected while optical density was monitored at 260 nm. Each fraction was precipitated with 1 ml of acetone for 1 h at 4°C and then centrifugated at 13 000 r.p.m. The protein pellets were re-suspended in 20 μl of SDS-sample buffer, separated by SDS-PAGE and then electroblotted to nitrocellulose membrane. The aIF2/5B protein was visualized by probing the membrane with antibodies anti-aIF2/5B and detected by ECL.

### *In vitro* translation and coexpression experiments

The *in vitro* translation reactions were performed according to described techniques (Condo *et al*., 1999). The samples contained the following in a final volume of 25 μl: Mix I: 10 mM KCl, 20 mM TEA-HCl, pH 7, 20 mM MgCl_2_, 7 mM β-mercatoethanol, 3 mM ATP, 1 mM GTP, 5 μg of bulk *S. solfataricus* tRNA, 2 μl of [^35^S]-methionine (1200 Ci mmol^−1^ at 10 mCi ml^−1^), 5 μl of *S. solfataricus* S30 extract containing about 4 pmol μl^−1^ ribosomes (pre-incubated for 10 min at 73°C) and 0.4 pmol μl^−1^*in vitro* transcribed mRNA.

The samples were incubated for 40 min at 73°C. At the end of the incubation 10 μl of the mixtures were withdrawn and electrophoresed on 15% acrylamide-SDS minigels. The radioactive bands were detected and quantified using either an Instant Imager apparatus (Packard) or an X-ray film.

*In vitro* translation of leadered and leaderless mRNAs in the presence of simultaneous coexpression of initiation factor mRNA was performed as described above, except that Mix I was supplemented with 0.4, 0.8 or 1.6 pmol μl^−1^ aIF2/5B mRNA. At the end of reaction, the [^35^S]-methionine-labelled proteins were separated on a 15% SDS-polyacrilamide gel. The gels were dried under vacuum and exposed to a Molecular Dynamics Phosphoimager screen for quantification.

### Binding of native and chimeric proteins to 30S and 50 ribosomal subunits

Each reaction was performed in 60 μl of 20 mM Tris-HCl pH 7.7, 7 mM Mg(OAc)_2_, 150 mM NH_4_Cl, 1 mM DTT and 5 μg per tube BSA, a fixed amount of proteins (50 pmol of IF2/5B, IF2, BaSu1 or BaSu2) and increasing amounts of 30S or 50S ribosomal subunits from *B. stearothermophilus* or *S. solfataricus*. After incubation at 37°C (55°C in the experiment with *S. solfataricus* ribosomes) for 10 min, 30 μl of 30% sucrose in the same incubation buffer was added and the samples were centrifuged for 30 min at 436 000 *g* (RC M120 GX Sorvall ultracentrifuge, rotor S100AT3) at 4°C. Fifteen microlitres of supernatant was withdrawn from the top of each tube and subjected to SDS-PAGE analysis followed by densitometric determination of the amount of protein remaining unbound.

### GTPase activity assay

Each reaction was performed in 50 μl of 25 mM Tris-HCl pH 7.8, 7 mM Mg(OAc)_2_, 70 mM NH_4_Cl, 30 mM KCl, 1 mM DTT, 10 pmol each of *B. stearothermophilus* or *S. solfataricus* 70S ribosomes, increasing amounts (5–30 pmol) of *B. stearothermophilus* IF2, *S. solfataricus* aIF2/5B or the chimeric factors, and 50 μM of a mixture of cold and [γ-^32^P]-GTP (10 Ci mmol^−1^). After incubation for 15 min at 55°C or 65°C, the extent of GTP hydrolysed was determined by isopropylacetate extraction in an acidic environment of the ^32^P_i_–dodecamolybdate complex as described by Parmeggiani and Sander (1981).

### tRNA protection assay

The interaction of the native and chimeric proteins with fMet-tRNA or Met-tRNA was investigated determining their ability to protect these tRNAs from the spontaneous hydrolysis occurring at alkaline pH (Gualerzi *et al*., 1991). Reaction mixtures, 50 μl in 100 mM Tris-HCl pH 8.0, 160 mM NH_4_Cl, 6 mM Mg(OAc)_2_ and 1 mM DTT, contained 10–15 pmol of tRNA (f^35^SMet-tRNA or ^35^SMet-tRNA) and 10–400 pmol of proteins as indicated in Fig. 6. Samples, withdrawn after 0 and 60 min of incubation at 37°C, were spotted on Whatman 3MM paper discs for determination of the cold trichloroacetic acid-insoluble radioactivity.

### Interaction of *S. solfataricus* Met-tRNAi with archaeal ribosomes

Increasing amounts (10–50 pmol) of recombinant aIF2/5B were incubated for 15 min at 65°C in 50 μl of 10 mM KCl, 20 mM TEA, 10 mM MgCl_2_ with 1 mM GTP, 100 pmol [35S]Met-tRNAi (specific activity ∼500 c.p.m. pmol^−1^), 20 pmol of *in vitro* transcribed mRNA 104 (Condo *et al*., 1999) and 20 pmol of *S. solfataricus* 70S ribosomes. The samples were immediately electrophoresed on non-denaturing 4% polyacrylamide gels made in 20 mM K(OAc), 2.5 mM MgCl_2_ and 40 mM Tris-HCl, pH 6, then dried and exposed to an X-ray film to visualize the labelled tRNAi.

### Interaction of fMet- and Met-tRNAi with bacterial ribosomes

Initiation complexes were prepared in 25 μl of 50 mM Tris-HCl pH 7.7, 7 mM Mg(OAc)_2_, 150 mM NH_4_Cl, 1 mM DTT, 1 mM GTP using 40 pmol of tRNAs (f^35^SMet-tRNA, ^35^SMet-tRNA), 25 pmol of mRNAs (AUG 022 mRNA) (La Teana *et al*., 1993), 40 pmol of ribosomes (*B. stearothermophilus* 30S) and increasing amount of native or chimeric proteins. After incubation at 37°C for 15 min, the amount of initiation complex formed was determined by nitrocellulose filtration (BA-85 filters, Schleicher and Schuell) followed by liquid scintillation counting.
